# Occurrence of Contralateral Lymph Neck Node Metastasis in Patients with Squamous Cell Carcinoma of the Oral Cavity

**DOI:** 10.4317/jced.51163

**Published:** 2014-06-01

**Authors:** Liziane C. Donaduzzi, Ferdinando De-Conto, Luana S. Kuze, Gisele Rovani, Mateus E. Flores, Adriano Pasqualotti

**Affiliations:** 1 Dentist graduated at the University of Passo Fundo, Brazil; 2 DDS Oral e Maxillo Facial Surgery, School of dentistry, University of Passo Fundo- RS, Brazil; 3 MSc Dentistry by University of Passo Fundo, Brazil; 4 MSc Oral Pathology, School of dentistry, University of Passo Fundo- RS, Brazil; 5 DDS Radiology, School of dentistry,University of Passo Fundo - RS, Brazil; 6 DDS Statistic, University of Passo Fundo – RS, Brazil

## Abstract

Introduction: Squamous cell carcinoma represents about 90% of malignancies of the mouth and about 38% of the head and neck tumors. The behavior of the cancer is very aggressive, presenting early cervical metastasis and, often contralateral ranging from 0.9 to 36%. Objectives: This study aims to analyze clinical and pathological factors that may influence metastasis of squamous cell carcinoma in cervical lymph nodes and relate this occurrence in the contralateral primary tumor, with disease prognosis and the interference of this type of metastasis in the survival rate of patients with this pathology. Material and Metohds: It was conducted a retrospective study from medical records of patients with Squamous Cell Carcinomas with homolateral lymph node metastasis and contralateral attended at the clinic of Head and neck surgery of Hospital São Vicente de Paulo in Passo Fundo – RS - Brazil, from 2000 to 2008. Results: Analyzing the charts of patients with metastatic and metastatic contralateral side it was observed that patients with initial stage presented a higher survival with statistical significance (p=0,035). Conclusions: The occurrence of metastases in lymph nodes of contralateral position to the primary lesion was not the main fator that influenced the survival of the group.

** Key words:**Squamous cell carcinoma, oral cavity, contralateral, death rate, lymphatic metastasis, prognosis.

## Introduction

Head and neck cancer is the fifth most common type of cancer worldwide, among all neoplasms. Approximately 40% of them occur in the oral cavity. Squamous cell carcinoma was the most common histological type, with a frequency observed in the literature approximately 90%. The overall survival rate is variable, depending on the primary site, disease stage and also the occurrence of metastasis, resulting in an unfavorable prognosis (1).

The squamous cell carcinoma of the oral cavity presents a variable frequency of contralateral metastases between 0.9% to 36%, reported in the literature. The presence of such metastases decreases the survival rate of the patients, on average, five years generating a poor prognosis (2).

Several clinical factors – pathological has been analyzed as predisposing to the development of contralateral lymph neck node metastasis [CLNM] of squamous cell carcinoma, such as: gender, location, extent, stage of primary tumor, positive ipsilateral lymph node involvement of midline, degree of histological differentiation, surgical margins, growth pattern, thickness and perineural invasion. The most significant prognostic factors are the place and size of the primary tumor and the variables relating to lymph node metastasis, including the length, number, location and transcapsular involvement. These factors, if found in a meaningful way, reduce the probability of local and cervical control of the disease and survival rates (3).

The contralateral metastasis propagation can occur in the head and neck carcinoma by: passage in afferent lymphatic vessels and tumor spread along the midline, which reaches the side efferent lymphatic vessels, when ipsilateral lymph nodes are widely involved, and in certain anatomical areas where there is not a real barrier in the middle line (2). The squamous cell carcinoma of the oral cavity has a high incidence of micrometastases and often bilaterally metastases due the rich submucosal lymphatic plexus, that communicates freely crossing the midline (4).

This study aims to analyze clinical and pathological factors that may influence metastasis of squamous cell carcinoma in cervical lymph nodes and relate this occurrence in the contralateral primary tumor, with disease prognosis and the interference of this type of metastasis in the survival rate of patients with this pathology.

## Material and Methods

It was performed a retrospective study obtained by histopathological findings in human tissue samples from 303 patients with Squamous Cell Carcinoma. These samples were selected among patients who were treated in the ambulatory of head and neck surgery of the Hospital São Vicente de Paulo in Passo Fundo – RS - Brazil and submitted to a surgical procedure of lesion resection before they are submitted or not to Radiotherapy and/or Chemotherapy. Besides, it was analyzed the medical records of each patient doing a survey of clinical and pathological factors common among cases, that can classify patients at high risk for metastasis of squamous cell carcinoma in the contralateral lymph nodes of the neck. Data about ethnicities, age, education level, smoking habit, alcohol consumption, topographic location of the inicial lesion and tumor stage were collected.

For the clinical evaluation of patients with the disease, TNM system was used, standardized by UICC and American Joint Committee on Cancer [AJCC] (5). However, for N and M, it was considered the information availa-ble in medical records and archived paraffin blocks. Patients were classified according to the presence or absence of metastasis in ipsilateral and contralateral lymph neck nodes.

For the histological tumor grading, it was used the OMS criteria, in which the tumors are classified as well, moderately or poorly differentiated according to the degree of histological differentiation (6). For this evaluation was used the information already contained in the pathology report held by the pathologist in charge of the Pathology Service of the Hospital São Vicente de Paulo in Passo Fundo - Brasil.

It was excluded from the study cases with insufficient material, patients whose medical record data were incomplete and all living patients or the date of death could not be analyzed were excluded from the study.

The collected data were analyzed by SPSS version 18 using parametric test [Kolmogorov-Smirnov] and non-parametric [Kruskal-Wallis; Mann-Whitney].

The project was approved by the Ethics Committee in Research of the Universidade de Passo Fundo and to the Hospital São Vicente de Paulo of Passo Fundo – RS, Brazil.

## Results

From 303 patients initially studied, 106 demonstrated lymph node involvement, in other words, in the TNM classification the N ≥ 1, which were subdivided into patients with ipisilateral or contralateral lymph neck node metastasis.

For analysis of the influence of metastasis in contralateral cervical lymph nodes on survival, it was discarded all patients who are alive and those who did not have the date of death for purposes of calculating the probability of the event [survival of the individual] throughout the period stipulated. The group with CLNM corresponded to 18.8% [n=15] and the group without CLNM 81.3% [n=65].

From the medical records of patients included in the analysis of the data of this research, the vast majority were men 93.8% [n=75] and only 6.3% [n=5] were women. The patients comprised only two ethnicities. White in 92.5% [n=74] and Browns in 7.5% [n=6].

The decade of life most affected by the condition studied was the 5th decade in 36.3% of cases [n=29], followed by 26.3% 4th decade [n=21]. The education level was analyzed, and 85% [n=68] that reported this data, the majority had finished high school 70.6% [n=48] followed by illiterate 11.8% [n=8], high school graduates 7.4% [n=5], primary school graduates 5.9% [n=4] and college graduates 4.4% [n=3].

The topographic location of the initial lesion was grouped and considered the following locations: mouth floor 18.8% [n=15], tongue 50% [n=40], vestibule 15% [n=12], palate 11.3% [n=9] and other locations 5% [n=4].

The tumor stage at diagnosis could not only be observed in the records of a single patient. The stage I was observed in 8.8% of patients [n=7], stage II in 66.3% [n=53], stage III in 22.5% [n=18] and stage IV 1.3% [n=1].

The treatment used in each case was described in all medical records and distributed in surgical procedure [50%, n=40], radiotherapy [8.8%, n=7], chemotherapy [5%, n=4], combination of these types of treatments [25%, n=20], no treatment [7.5%, n=6], and other types of treatments performed [3.8%, n=3]. Among the treatments used in patients with CLNM, the surgical corresponded to 80% [n=12] and other forms of treatment 20% [n=3]. From the patients with lateral lymph node involvement 44.6% [n=29] had surgical treatment and 55.4% [n = 36] other types of treatments.

Smoking was informed by 85% [n = 68] of the medical records and was present in the habits of 91.2% [n = 62] of patients. Alcohol consumption was informed by 80% [n = 64] of patients, 90.6% [n=58] drinking ( Table 1). These data were not crossed with survival due to the large number of missing data.

**Table 1:**
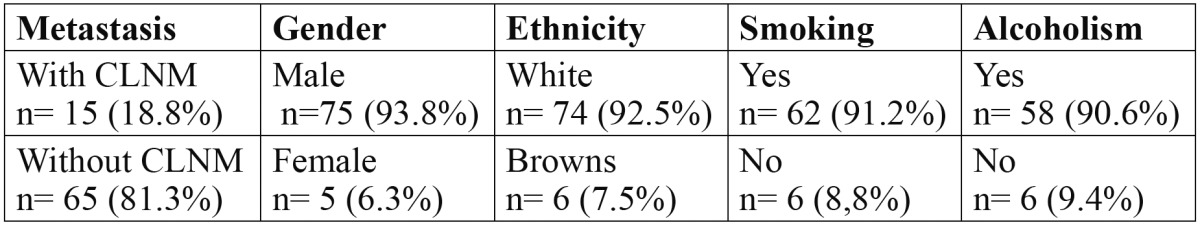
Prevalence of clinical aspects.

**Figure 1:**
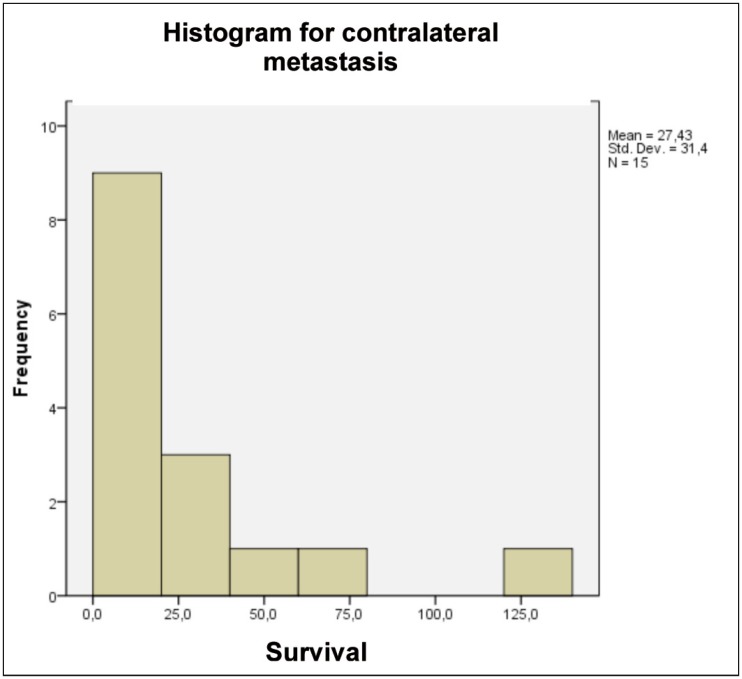
Histogram of the frequency of overall survival (months) of patients with contralateral metastasis who died from cancer.

It was performed the test of the survival curve and using the histogram analysis of patients with CLNM. The average survival was 17 months (Fig. 1). There was no statistically significant difference in survival of patients with ipsilateral lymph neck node metastasis and those with contralateral lymph neck node metastasis of site tumor origin [p=0,086].

**Figure 2:**
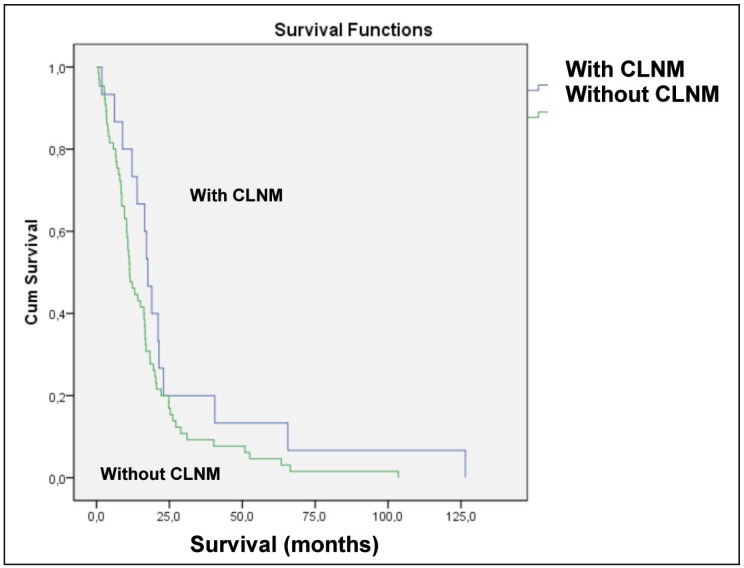
Graphic of the frequency of patients with CLNM and without CLNM with survival in months.

Survival probability begins to decrease regardless to have or not CLNM, from 65-70 months, and there is a 50% chance of dying up to 50 months, more than 60% of patients have a chance of dying before 2 years (Fig. 2).

Later at intersections, the variables that lacked information or classification were discarded and some data were adjusted for statistical purposes.

**Figure 3:**
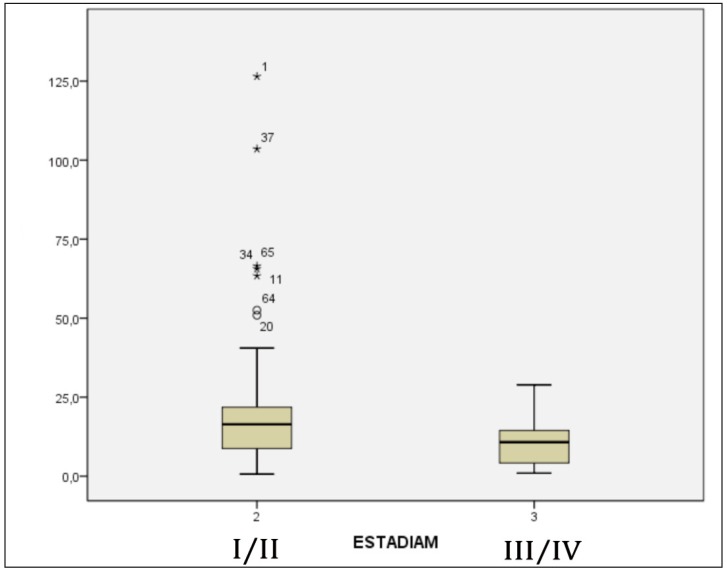
Graphic of the interval of survival in months of the patients who died, according to staging.

**Figure 4:**
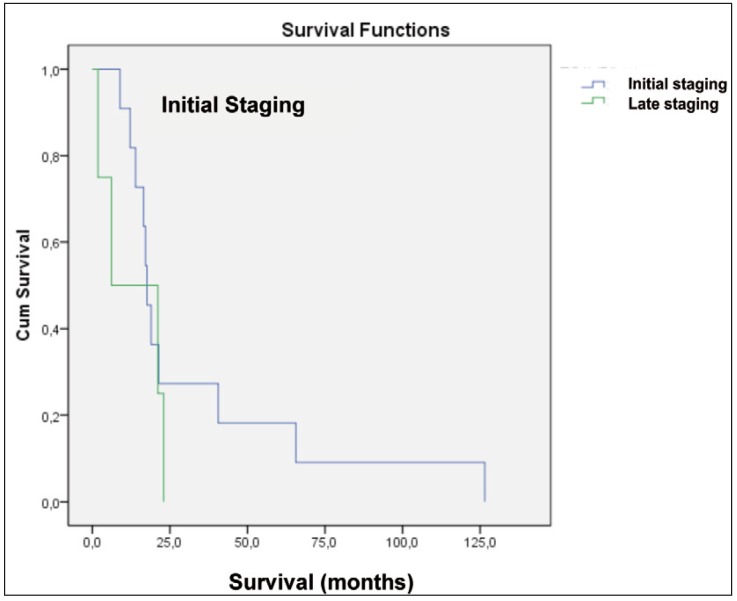
Graphic of survival in months of patients with contralateral lymph node involvement who died, according to the staging.

The staging was classified in initial, including patients with stages I and II, and late, patients with stages III and IV. In analyzing the graphics of the patients with and without CLNM it was possible to notice that patients with initial staging showed a higher survival with statistical significance [p=0,035] (Fig. 3,4).

Of the patients with contralateral lymph neck node involvement, 73.4% [n=11] were classified in initial staging at diagnosis, with a median survival of 32.6 months. Patients with late staging 26.6% [n = 4] had an average of 13.0 months.

**Figure 5:**
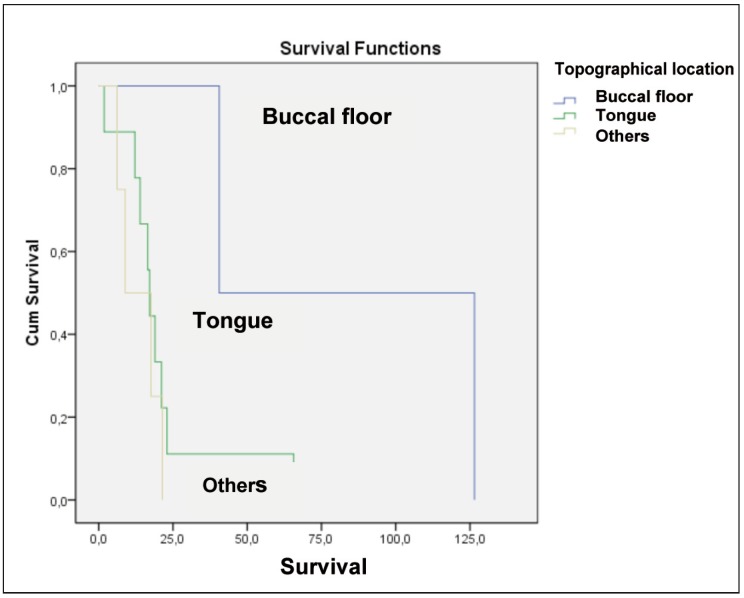
Graphic of survival in patients with contralateral lymph node involvement who died according to the topographical location of injury diagnosis.

In comparison of each site in relation to metastasis, it was noted that among patients who had compromised contralateral lymph nodes and had initial tumor in floor demonstrated greater survival (Fig. 5).

## Discussion

In the study, patients with CLNM corresponded to the smallest group with 18.8% (n = 15) and patients without CLNM corresponded to 81.3% (n = 65), similar to the previous study, where the CLNM group was also lower (7).

There was a predominance of males [93.8%, n = 75] and only 6.3% [n = 5] were women agreeing with results of other studies (7,8). The highest incidence of squamous cell carcinoma occurs in the age group above 45 years old, with a higher prevalence in males and white people (1,9,10). Researchers believe with age increases the risk of developing cancer of the oral cavity by aggregating harmful effects of carcinogens (10). In the study the most affected decade of life by carcinoma was the 5th decade in 36.3% of cases [n=29]. Analyzing race, white patients were also more affected [92.5%, n=74].

Oral squamous cell carcinoma can occur anywhere in the mouth, being the most affected sites the tongue, lower lip and buccal floor. These regions are greatly facilitators of the carcinoma spreading to regional lymph nodes and / or distant organs (11). The most affected segment by injury in both groups was the tongue [50%, n=40] followed by the floor of mouth [18.8%, n = 15], similar to that found in the literature. The topographical location of the lesion showed no statistical influence on survival, as well as of other studies (12,13).

In the group with CLNM 60% [n=9] presented initial injury in tongue and 13.4% [n=2] in floor of mouth. The clinical stage, tumor comprising the midline and involvement of floor of mouth, were the main factors predisposing to CLNM. Patients with primary tumors in floor of the mouth had a 50% higher risk of contralateral metastasis than those with tumors in tongue (14). In the study, patients with initial tumor in the tongue represented 60% of the group with contralateral lymph node involvement.

The advanced clinical TNM and tumor size can be considered important factors that favor the development of CLNM, being as important as the location of the tumor and involvement midline (7). The stage which the tumor is at diagnosis shows itself a very important factor when observed patient survival. Metastatic lymph nodes represent one of the most relevant aspects associated with treatment, characterizing an advanced clinical stage [stages III or IV], and it is associated with a 50% reduction in survival (15). This study shows that patients with CLNM and late staging live an average of 13.0 months, while in the initial staging 32.6 months. When analyzed patients who were diagnosed in the early stages of the disease, there was a survival in months higher than those diagnosed at later stages.

Analyzing both groups together [n=80], it was observed that patients with initial stage showed a significantly higher survival [p=0,035]. When analyzing the separate groups, could still be observed difference in survival of patients with initial staging from those with late staging. Therefore, metastasis was not the main influencing factor on the survival of the group with contralateral lymph node involvement.

Neck treatment with metastatic is the radical dissection, contemplating possible changes related to the preservation of non-lymphatic structures. In view of the high rates of occult metastases ipsilateral and contralateral neck in an untreated neck and the poor salvage rates for failures at the neck, elective neck treatment of the N0 neck had been proposed by investigators as reported in the literature (16). Among the treatments performed, the radical surgical procedure was the most recommended, followed by combining this with radiotherapy or chemotherapy either in the analysis of both groups. The combination of concurrent chemotherapy and radiotherapy has become standard treatment for many patients with improved locoregional control and overall survival when compared with radiotherapy alone (17), justifying the small percentage of this treatment option, being more used when the patient has no clinical conditions to undergo surgery.

Analyzed studies have reported that the recurrence of the primary lesion and the presence of metastasis in cervi-cal lymph neck nodes contralateral affect the survival rate of patients with squamous cell carcinoma of the oral cavity [CCE], thereby generating, an unfavorable prognosis (2).

When comparing the survival of patients with CLNM with patients without CLNM, there was no statistical difference between the groups, agreeing with results of another study (18). However, the probability starts decreasing regardless of whether or not to have metastasized, from 65-70 months, and there is a 50% chance of dying up to 50 months, more than 60% of patients are likely to die before 2 years, similar to the findings of the literature reporting high probability of dying before age 5 (3,7). It should be remembered that all patients who are part of the study, already had lymph neck node metastasis, however, some ipisilateral and others contralateral, this already means an advanced stage of the disease. Study reported not finding a poor prognosis in patients with metastasis in a single lymph node and contralateral metastases limited to levels 1 and 2 (3). In the study there were no data about the amount and level of contralateral metastatic lymph nodes, hypothesis that could have generated no statistical significance in survival between the groups.

The occurrence of lymph node metastasis of contralateral position to the primary lesion was not the main factor that influenced the survival of the group. It is necessary emphasizes the importance of early diagnosis, preventing squamous cell carcinoma to reach an advanced stage.
